# The joint association of physical activity and sedentary behavior with metabolic syndrome among urban men aged 60+ years in regional China

**DOI:** 10.3389/fpubh.2022.1073000

**Published:** 2022-11-24

**Authors:** Qinglin Lou, Haidi Wu, Guang Li, Yan Hu, Qing Ye, Shouyong Gu, Fei Xu

**Affiliations:** ^1^Department of Endocrinology, Geriatric Hospital of Nanjing Medical University, Nanjing, China; ^2^Department of Endocrinology, Jiangsu Province Official Hospital, Nanjing, China; ^3^Department of Laboratory Medicine, Geriatric Hospital of Nanjing Medical University, Nanjing, China; ^4^Department of Laboratory Medicine, Jiangsu Province Official Hospital, Nanjing, China; ^5^Nanjing Municipal Center for Disease Control and Prevention, Nanjing, China

**Keywords:** metabolic syndrome, elderly men, physical activity, sedentary behavior, exercise

## Abstract

**Objectives:**

Metabolic syndrome (MetS) is a major public health issue worldwide, which is preventable through physical activity (PA) promotion and sedentary behavior (SB) reduction. However, the joint association of PA and SB with MetS was not well-investigated, particularly in elderly people. This study aimed to examine separate and joint associations of PA and SB with MetS among elderly urban men in China.

**Methods:**

In this cross-sectional study conducted in mid-2018, participants were urban men aged 60+ years randomly selected from in Nanjing of China. Exposure variables were PA and SB. The outcome variable was MetS. A participant was categorized as “having MetS” or “not having MetS” in the analysis. Independent variables were PA and SB, which were categorized as “sufficient PA or insufficient PA” and “shortened SB or prolonged SB”, respectively. Mixed-effects logistics regression models were applied to calculate odds ratios (ORs) and 95% confidence intervals (CIs) to assess the association of PA and SB with MetS.

**Results:**

Totally, 5,520 from 5,792 eligible participants were randomly recruited and their mean age was 68.9 (standard deviation: 16.9) years. The prevalence of MetS was 30.8% (95%CI = 29.6%, 32.0%) among urban men aged 60+ years in the study. After adjustment for potential confounders, subjects with sufficient PA were less likely (OR = 0.77, 95%CI = 0.67, 0.88) to experience MetS, independently of SB, relative to their counterparts with insufficient PA, while a lower odds (OR = 0.74; 95%CI = 0.61, 0.89) of experiencing MetS was examined for participants with shortened SB, also independently of PA, compared to those with prolonged SB in the study. Furthermore, compared to participants with insufficient PA and prolonged SB, those either within categories of insufficient PA and shortened SB (OR = 0.81; 95%CI = 0.65, 0.99), sufficient PA and prolonged SB (OR = 0.80; 95%CI = 0.70, 0.92), or sufficient PA and shortened SB (OR = 0.41; 95%CI = 0.26, 0.63) were at significantly lower risk to experience MetS, respectively.

**Conclusions:**

PA was negatively associated with MetS, and SB was positively linked to MetS, which were independent of each other. Moreover, sufficient PA and shortened SB might exert additively joint influence on MetS. This study has important implications that concurrent PA promotion and SB reduction shall be encouraged for people to optimize the effectiveness of MetS prevention.

## Introduction

Metabolic syndrome (MetS) is a complex metabolic concept, which is characterized typically by the concurrence of at least three of the following cardio-metabolic conditions: abdominal obesity (AO), increased fasting blood glucose (increased-FBG), raised blood pressure (raised-BP), elevated triglycerides (elevated-TG), and/or lowered high-density lipoprotein cholesterol (lowered-HDL-C) (1). It has been examined that individuals with MetS are at high risk of diabetes, cardiovascular and cerebral diseases, some cancers and neurodegenerative diseases (2–4). As different identification criteria of these five MetS components were suggested for ethnic-specific population, there is no globally uniform diagnostic criterion of MetS, (5). Currently, three widely-used MetS definitions for population-based surveys were recommended by World Health organization (6), National Cholesterol Education Program (7) and International Diabetes Federation (1, 8). However, regardless of different diagnostic criteria of MetS, it has been estimated that the prevalence of MetS was ~25% for adult population worldwide (9), and one-third and 24.5% among adults, respectively, in USA (10) and China (11). Therefore, from the perspective of population health, MetS is a major issue worldwide and a priority of public health concern.

Fortunately, MetS and its components are preventable through lifestyle and behavior intervention, particularly physical activity (PA) promotion or/and sedentary behavior (SB) reduction campaigns (12). In both developed and developing societies, PA has been examined to be negatively associated with MetS (10, 13–15), while SB was in positive relation to MetS (10, 16–18). Among those studies on the relationship between PA, SB and MetS, the majority reported the separate association of PA, SB with MetS, and very few documented the combined relationship between PA, SB and MetS (13–17). To maximize the output-input ratio, PA promotion and SB reduction were encouraged to be integrated into a single intervention campaign for prevention of chronic diseases including MetS and its components. Thus, it is of particular interest to well understand the joint association of PA and SB with MetS in addition to the separate relationship between them.

MetS was examined to be highly age-dependent, as its prevalence was observed consistently higher among the older than the younger across different ethnic sub-populations worldwide (19). Meanwhile, elderly people have more discretionary time in daily life relative to their younger counterparts, as they, particularly urban residents, typically get retired and no longer have to do jobs. So, elderly people bear a heavy burden of MetS and its components, and, on the other hand, they have sufficient leisure time. Consequently, elderly people are the priority population for MetS prevention through leisure-time PA promotion and/or sedentary behavior reduction. In China, the prevalence of MetS increased from 13.7% in 2000 to 24.5% in 2015 among adult population (12, 20), while the proportion of elderly people aged 60+ years (the mandatory age for retirement) increased from 10.4% in 2000 to 18.7% (about 260 million) in 2020 and the urbanization rate increased from 36.0% in 2000 to 63.9% (~902 million) in 2020 (21). Clearly, China has been witnessing an alarming increase in a multi-burden caused by MetS, rapid aging of population and urbanization over the past two decades. Thus, it is of public health significance to well investigate the joint association of PA and SB with MetS among elderly people, particularly those urban retirees, in China, as it is important for community-based MetS prevention through precision intervention of leisure—time PA and SB among older residents.

To date, there was a lack of studies that documented the combined relationship between PA, SB and MetS among elderly people in urban areas of China. To fill this gap, a population-based study was conducted among urban men aged 60+ years in Nanjing municipality of China, with aims to examine whether: (1) PA and SB were associated with MetS independent of each other, and (2) an additive effect of sufficient PA and shortened SB on MetS existed.

## Methods

### Study design and participants

A cross-sectional survey was conducted initially for estimating the prevalence of diabetes and hypertension comorbidity among urban elderly men during mid-2018 in Nanjing, a typical mega-city in eastern region of China (DiaHyCom study). The eligible participants referred to those locally registered urban residents who: (1) were men aged 60+ years, (2) were without physical disability or/and psychiatric disorders, and (3) had no cognitive/literal problems. Participants were randomly chosen using a multi-stage sampling approach. The sample size was determined with consideration of the sampling method, estimated prevalence (7.5%) of diabetes and hypertension comorbidity among elderly people in China (22), and the expected statistical power (90%). Thus, ~4,500 participants would be sufficient for DiaHyCom study.

**Figure 1:**
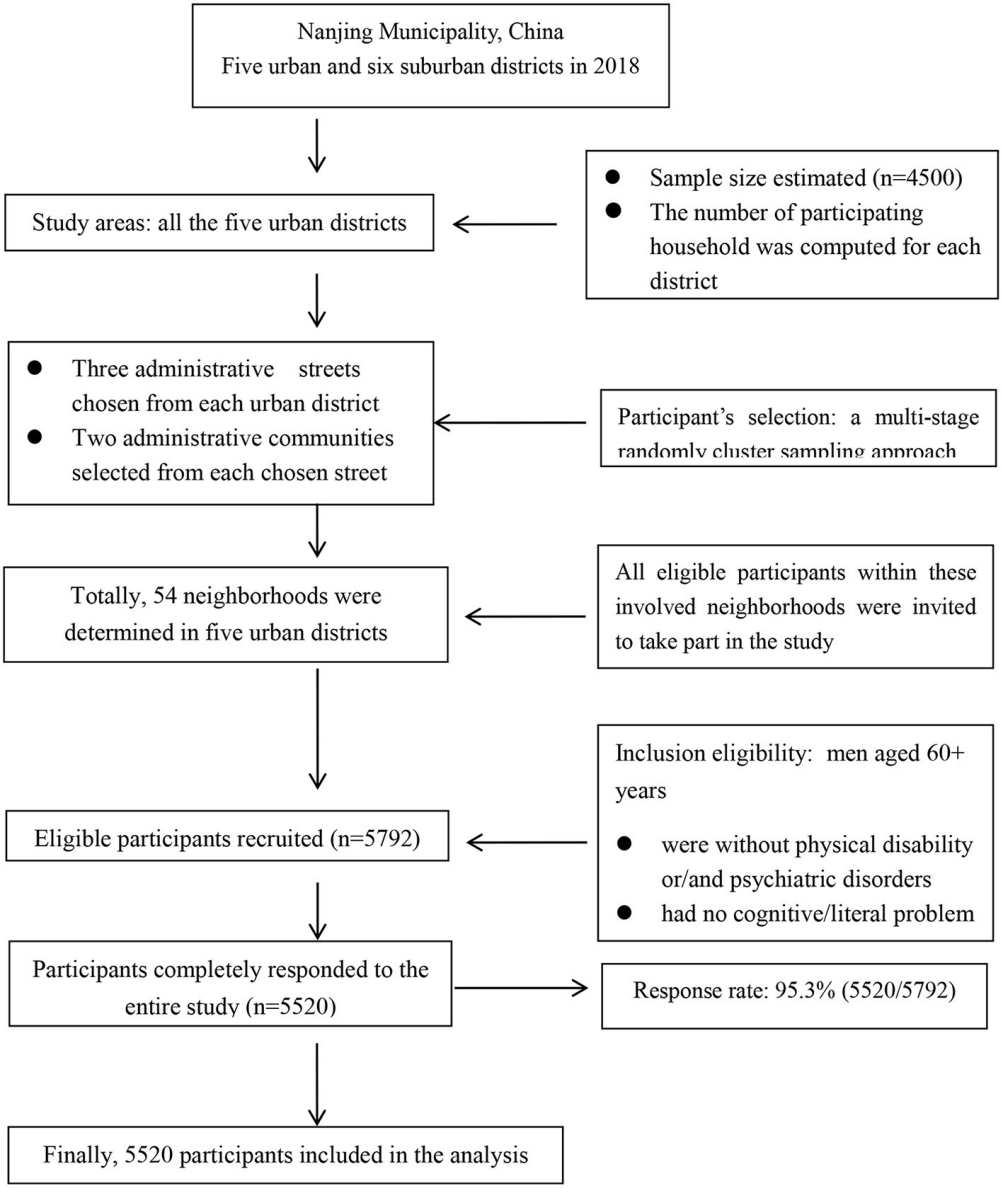
Flow chart of participant's selection in the study.

Currently, China has a five-stratum administrative system, including central government, province/municipality, urban district/rural country, administrative street/town and administrative urban community/rural village. Each administrative community or village is usually composed of different numbers of neighborhoods. In Nanjing, there were totally five urban and six suburban districts in 2018. For recruitment of participants in the study, all the five urban districts were included and the sampling unit was neighborhood. Prior to selection of participants, two figures were estimated: (1) a specific number of participants was calculated for each district based on the proportion of elderly population of the district to the overall of all five urban districts; and (2) with the assumption that, on average, one household had one elderly man aged 60+ years, the number of participating household was computed for each district. Then, a multi-stage sampling approach was used to randomly select participants from each district. Firstly, three administrative streets were randomly chosen from each of the five urban districts. Secondly, two administrative communities were randomly selected from each chosen street. Thirdly, neighborhoods were randomly determined from each involved administrative community according to the number of participating households, resulting in a total of 54 neighborhoods selected. Finally, all eligible men aged 60+ years within each involved neighborhood were invited to take part in the study. Participant's selection flowchart was shown in Figure 1.

Written informed consents were obtained from all participants before the survey. The data analyzed to examine the relationship between PA, SB and MetS in the present study were derived from DiaHyCom survey and de-identified before analysis. Thus, second-hand data were analyzed in this study, which was also reviewed by The Ethics Committee of Geriatric Hospital of Nanjing Medical University. The methods performed in the present study were in accordance with relevant recommendations by the Declaration of Helsinki.

### Data collection

Information on participant's socio-demographic characteristics (including age, educational attainment, marital status, etc), history of major chronic diseases, family history of diabetes and hypertension, physical activity and sedentary behavior, cigarette smoking, drinking, consumption of meat were gathered *via* a standardized questionnaire (23). All the information was self-reported by participants *via* a face-to-face interview which was administered by our research team members.

Data on each participant's blood pressure and anthropometry were objectively measured. Blood pressure was assessed with calibrated sphygmomanometers based on the Chinese guidelines for blood pressure measurement (24). In the guidelines, Korotkoff sounds were used to determine participant's blood pressure with a standardized procedure (24). Moreover, at least two readings were recorded and the mean value was used for analysis (24). With regard to anthropometric measurement, all participants were asked to stay in a quiet room for a minimal 5-min rest prior to measurement. Waist circumference (WC) was measured to the nearest 0.1 cm at the midpoint between costal inferior and iliac crest for each participant who was with light indoor clothing (25). WC was also recorded twice and the mean value was used for each participant in the analysis.

For assessing the levels of blood glucose, triglyceride and high density lipoprotein cholesterol, a 5-ml fasting venous blood sample was collected from each participant. All the blood samples were sent to the laboratory of a designated hospital for analysis by well-trained staff. The automatic biomedical analysis instrument was HITACHI7180 analyzer (Hitachi Co., Japan) and detection kits were from Shanghai Fosun Long March Medical Science Co., China.

### Study variables

#### Outcome variable

The outcome variable was MetS, which was defined based on the diagnostic criteria recommended specially for Chinese adults by Chinese Diabetes Society (CDS) in 2017 (26). A participant was categorized as “having MetS”, if he concurrently experienced at least three of the following abnormal health conditions: abdominal obesity (WC ≥ 90 cm) for a man, increased fasting blood glucose (FBG ≥6.1 mmol/L, and/or diagnosed with diabetes), raised blood pressure (BP ≥130/85 mmHg, and/or diagnosed with hypertension), elevated triglycerides (TG ≥1.70 mmol/L), and/or lowered high-density lipoprotein cholesterol (HDL-C < 1.04 mmol/L). Otherwise, a participant was classified as “not having MetS”.

#### Independent variables

There were two explanatory variables, PA and SB, in this study. Both of them referred to leisure-time activities and were assessed using the validated Chinese version of International Physical Activity Questionnaire (IPAQ-CHN) (27, 28). This IPAQ-CHN was professionally translated from the original IPAQ and has been validated for Chinese adults (28, 29). For each participant, PA and SB time in the last seven days was self-reported using IPAQ-CHN. The weekly time of moderate and vigorous PA was recorded separately. Then, based on the sum of weekly moderate PA time plus doubled vigorous PA time (moderate and vigorous physical activity, MVPA), a participant was classified into the sub-group of “sufficient PA (≥150 min/week)” or “insufficient PA (<150 min/week)” in the analysis (29, 30). On the other hand, daily sedentary behavior time was defined as screen viewing time, which was assessed with a question asking each participant about the time he spend sitting each day for screen viewing in the last 7days. Then, SB time was also computed and used to categorize participants into: “shortened SB (<2 h/day)” or “prolonged SB (≥2 h/day)” according to SB time recommendations for Chinese people (29).

In addition to separate relationship between PA, SB and MetS examined in the present study, the combined association of PA and SB with MetS was also investigated. Thus, participants were further classified into one of the following sub-groups: insufficient PA and prolonged SB (the reference with the highest risk), insufficient PA and shortened SB, sufficient PA and prolonged SB, or sufficient PA and shortened SB (the lowest risk group).

#### Covariates

Some classical covariates were controlled for in the analysis. They were age (younger: 60–69, middle: 70–79 or older: 80+ years), educational attainment (≤ 9, 10–12 or 13+ schooling years), cigarette smoking, alcohol drinking, consumption of meat, family histories of diabetes and hypertension.

A participant was defined as a current smoker, if he smoked one or more cigarettes every day for at least 1 year or smoked 18+ packs per year in total (25). If a person had quit smoking for at least 1 year, he was categorized as an ex-smoker (25). Otherwise, he would be classified as a non-smoker (25). In this study, smokers referred to both current smokers and ex-smokers. Drinkers were defined as persons who drank alcohol, on average, at least two times a week for more than 1 year, while non-drinkers were those people who did not meet drinker's criterion in this study (25).

Dietary consumption was measured with a validated Chinese version of food frequency questionnaire (FFQ) (31). The weekly frequency of meat intake was used for analysis in the study. The frequency of meat consumption, at least seven times per week on average, was recommended for Chinese old people by Chinese Nutrition Society (32). Thus, participants were categorized into two sub-groups based on whether or not they reached this meat intake recommendation: reached recommendation (“Yes”) or not reach recommendation (“No”).

A participant was defined as having a positive family history (“Yes”) of diabetes or hypertension, if at least one of his parents had been identified as diabetic or hypertensive patient, respectively. Otherwise, a participant was classified as not having a positive family history (“No”).

#### Data analysis

Descriptive analysis was implemented to present participant's characteristics (%). Differences in personal characteristics between age, PA, SB and MetS were, separately, compared with chi-square test. And, the differences in mean values (standard deviation, SD) of age, WC, FBG, BP, TG and HDL-C were examined using ANOVA approach. Then, two mixed-effects logistic regression models were employed to compute odds ratios (ORs) and 95% confidence intervals (CIs) for investigating the separate and joint associations of PA and SB with MetS. Model 1 was a univariate logistic regression analysis with PA, SB or their joint category as the single independent variable. Model 2 was a multivariate logistic regression analysis with PA, SB or their joint category as the independent variable and with adjustment for age, educational attainment, smoking, drinking, meat consumption, PA (where applicable), SB (where applicable), family history of diabetes and hypertension. In both mixed-effects logistic regression models, neighborhood-level potential clustering effects were considered as the random effect. Two-sided statistical significance level was set at *p* < 0.05. Data were entered with EpiData 3.1 (The EpiData Association 2008, Odense, Denmark) and analyzed using SPSS version 20.0 for Windows (SPSS Inc., Chicago, IL, USA).

## Results

### Selected participants' characteristics

Although the estimated sample size was 4,500, the actually chosen participants were 5,792 elderly men from 54 neighborhoods due to the cluster sampling approach applied in this study. Among those 5,792 participants, 5,520 (95.3%) completely responded to the entire study, including questionnaire survey, anthropometric assessment and blood sample collection. There was no difference in age and education level between those completed and did not complete the study. Table 1 displayed the selected characteristics of participants by age in this study. The majority (63.9%) of participants obtained educational attainment of 10–12 schooling years. And, the proportion of participants with 10–12 schooling years was 69.2, 57.3 and 44.3 for subjects aged 60–69, 70–79, and 80+ years, respectively (*p* < 0.001). The mean value of MVPA time was 89.8 (SD = 147.1) minutes in last week, while the mean sedentary behavior time was 4.1 (SD = 2.5) hours per day among the overall participants.

**Table 1:** Selected characteristics of participants by age in this study.

		**All participants**		**60-69 years**		**70-79 years**		**80**+ **years**		*p*-value^*^
		***N***	**%/Mean (±SD)**	***N***	**%/Mean (±SD)**	***N***	**%/Mean (±SD)**	***N***	**%/Mean (±SD)**	
Overall		5,520	100.0	3,518	63.7	1,611	29.2	391	7.1	
**Educational attainment (schooling years)**										
	0-9	994	18.0	560	15.9	331	20.5	103	26.3	<0.001
	10-,12	3,530	63.9	2,435	69.2	922	57.3	173	44.3	
	13+	996	18.1	523	14.9	258	22.2	115	29.4	
**Moderate and doubled vigorous PA time (minutes in last week)**										
	Mean (±SD)	5,520	89.8 (147.1)	3,518	92.2 (147.4)	1,611	90.4 (150.3)	391	65.2 (127.6)	<0.01
**Sedentary behavior time (hours per day)**										
	Mean (±SD)	5,520	4.1 (2.5)	3,518	4.2 (2.5)	1,611	4.0(2.4)	391	3.9 (2.3)	<0.01
**Smoking** ^**a**^										
	No	3,187	57.7	1,801	51.2	1,080	67.0	306	78.3	<0.001
	Yes	2,333	42.3	1,717	48.8	531	33.0	85	21.7	
**Drinking** ^**b**^										
	No	3,257	59.0	1,864	53.0	1,085	67.3	308	78.8	<0.001
	Yes	2,263	41.0	1,654	47.0	526	32.7	83	21.2	
**Meat consumption (reached recommendation)** ^**c**^										
	No	4,331	78.5	2,660	75.6	1,340	83.2	331	84.7	<0.001
	Yes	1,189	21.5	858	24.4	271	16.8	60	15.3	
**Family history of hypertension** ^**d**^										
	No	3,751	68.0	2,274	64.6	1,159	71.9	318	81.3	<0.001
	Yes	1,769	32.0	1,244	35.4	452	28.1	73	18.7	
**Family history of diabetes** ^**e**^										
	No	4,948	89.6	3,079	87.5	1,490	92.5	379	96.9	<0.001
	Yes	572	10.4	439	12.5	121	7.5	12	3.1	

* Chi-square test.

a Smoking status was defined as smokers (current- and ex-smokers) and non-smokers (who never smoked cigarettes).

b Drinkers were defined as persons who drank alcohol, on average, at least two times a week for more than one year, while non-drinkers were those people who did not meet drinker's definition.

c Meat consumption was classified as “reached recommendation” and “not reach recommendation” based on the frequency of meat consumption recommended for Chinese old people by Chinese Nutrition Society.

d Family history of hypertension was categorized as having a positive family history (“Yes”) of hypertension, if at least one of parents had been identified as hypertensive patient.

e Family history of diabetes was classified as having a positive family history (“Yes”) of diabetes, if at least one of parents had been identified as diabetic patient.

### Prevalence of PA, SB and MetS among participants

Table 2 presented the prevalence of PA, SB and MetS by selected characteristics of participants in the study. The proportion of participants with sufficient PA and prolonged SB was, separately, 26.9% (95%CI = 25.7, 28.1%) and 87.5% (95%CI = 86.6, 88.4%) in the study. The overall prevalence of MetS was 30.8% (95%CI = 29.6, 32.0%) among urban men aged 60+ years in the present study, while the stratified prevalence of MetS significantly differed among participants aged 60–69 years (29.7%; 95%CI = 28.2, 31.3%), 70–79 years (33.2%; 95%CI = 30.5, 36.0%) and 80+ years (30.7%; 95%CI = 26.2, 35.6%) in the study. Moreover, the difference in MetS prevalence was examined also significant between education, drinking, family histories of diabetes and hypertension.

**Table 2:** The proportion of participants by physical activity, sedentary time and metabolic syndrome among urban men aged 60+ years in regional China.

		**N of participants**	**Participants with sufficient PA** ^**a**^		*p*-value^*^	**Participants with prolonged SB** ^**b**^		*p*-value^*^	**Participants with MetS** ^**c**^		*p*-value^*^
			**%**	***n***		**%**	***n***		**%**	***n***	
Overall		5,520	26.9	1,485		87.5	4,830		30.8	1,699	
**Age (years)**											
	60–69	3,518	28.3	996		87.4	3,074		29.7	1,044	
	70–79	1,611	26.0	419	<0.001	87.8	1,414	0.93	33.2	535	0.04
	80+	391	17.9	70		87.5	342		30.7	120	
**Educational attainment (schooling years)**											
	0-9	994	23.2	231		74.2	738		25.7	255	
	10-12	3,530	27.2	959	<0.01	89.0	3143	<0.001	32.3	1,141	<0.001
	13+	996	29.6	295		95.3	949		30.4	303	
**Smoking** ^**d**^											
	No	3,187	25.6	816	0.01	88.0	2,806	0.15	29.9	953	0.10
	Yes	2,133	28.7	669		868	1,824		32.0	746	
**Drinking** ^**e**^											
	No	3,257	25.4	828	<0.01	87.0	2,932	0.14	29.7	968	0.04
	Yes	2,263	29.0	657		88.3	1,998		32.3	731	
**Meat consumption (reached recommendation)** ^**f**^											
	No	4,331	27.8	1,204	<0.01	86.9	3,765	0.02	30.6	1326	0.62
	Yes	1,189	23.6	281		89.6	1065		31.4	373	
**Family history of hypertension** ^**g**^											
	No	3,751	26.3	987	0.15	86.7	3,253	0.01	27.6	1,035	<0.001
	Yes	1,769	28.2	498		89.1	1,577		37.5	664	
**Family history of diabetes** ^**h**^											
	No	4,948	26.6	1,316	0.13	87.2	4,313	0.03	29.5	1,458	<0.001
	Yes	572	29.5	169		90.4	517		42.1	241	

* Chi-square test.

a Physical activity was categorized into “insufficient PA (<150 min/week)”and “sufficient PA (≥150 min/week)” based on weekly moderate physical activity time.

b Sedentary behavior was classified as “shortened SB time (<2 h/day)” or “prolonged SB time (≥2 h/day)” according to SB recommendations for Chinese people.

c MetS was identified according to diagnostic criteria recommended by Chinese Diabetes Society.

d Smoking status was defined as smokers (current- and ex-smokers) and non-smokers (who never smoked cigarettes).

e Drinkers were defined as persons who drank alcohol, on average, at least two times a week for more than one year, while non-drinkers were those people who did not meet drinker's definition.

f Meat consumption was classified as “reached recommendation” and “not reach recommendation” based on the frequency of meat consumption recommended for Chinese old people by Chinese Nutrition Society.

g Family history of hypertension was categorized as having a positive family history (“Yes”) of hypertension, if at least one of parents had been identified as hypertensive patient.

h Family history of diabetes was classified as having a positive family history (“Yes”) of diabetes, if at least one of parents had been identified as diabetic patient.

### Distribution of age, WC, FBG, BP, TG and HDL-C by PA and SB among participants

Table 3 demonstrated the distribution of age, WC, FBG, BP, TG and HDL-C among overall and stratified participants by PA and SB. For overall participants, their mean age (SD) was 68.9 (16.9) years, while the mean value (SD) of WC, FBG, systolic and diastolic BP, TG and HDL-C was, respectively, 87.2 (9.1) cm, 6.0 (1.7) mmol/L, 134.2 (16.3) mmHg, 80.7 (9.6) mmHg, 1.6 (1.2) mmol/L and 1.37 (0.50) mmol/L. Moreover, the mean value of age, WC and systolic BP each differed in participants with insufficient and sufficient PA, and mean values of WC, systolic and diastolic BP, and TG were different between subjects with shortened and prolonged SB, separately.

**Table 3:** Distribution of age, WC, FBG, BP, TG and HDL-C by PA and SB among urban men aged 60+ years in regional China.

		**Overall participants (N = 5,520)**	**Mean value (SD)** ^*^					***p*-value^#^**
			**PA** ^**†**^		***p*-value^#^**	**SB** ^**‡**^		**'**
			**Insufficient**	**Sufficient**		**Shortened**	**Prolonged**	
Age (years)		68.9 (16.9)	69.2 (16.9)	68.2 (6.1)	0.02	68.8 (6.7)	69.0 (15.6)	0.73
WC (cm)^a^		87.2 (9.1)	87.4 (9.2)	86.5 (8.6)	<0.01	86.3 (9.4)	87.3 (9.0)	<0.01
FBG (mmol/L)^b^		6.0 (1.7)	6.0 (1.7)	6.0 (1.7)	0.74	5.9 (1.7)	6.0 (1.7)	0.16
BP (mmHg)^c^							
	Systolic BP	134.2 (16.3)	134.5 (16.8)	133.3 (14.8)	0.02	137.0 (17.7)	133.0 (16.8)	<0.01
	Diastolic BP	80.7 (9.6)	80.9 (9.7)	80.4 (9.2)	0.15	82.2 (10.4)	80.5 (9.5)	<0.01
TG (mmol/L)^d^		1.6 (1.2)	1.6 (1.2)	1.6 (1.1)	0.19	1.5 (1.0)	1.6 (1.2)	<0.01
HDL-C (mmol/L)^e^		1.37 (0.50)	1.37 (0.51)	1.36 (0.48)	0.70	1.36 (0.40)	1.37 (0.51)	0.76

* SD: standard deviation.

# Differences in age, WC, FBG, BP, TG and HDL-C were examined with ANOVA approach.

† PA, Physical activity was categorized into “insufficient PA (<150 min/week)”and “sufficient PA (≥150 min/week)” based on weekly moderate physical activity time.

‡ SB: Sedentary behavior was classified as “shortened SB time (<2 h/day)” or “prolonged SB time (≥2 h/day)” according to SB recommendations for Chinese people.

a WC, Waist circumference.

b FBG, Fasting blood glucose.

c BP, Blood pressure.

d TG, Triglycerides.

e HDL-C, High-density lipoprotein cholesterol.

### Separate and joint associations of PA and SB with MetS

Table 4 showed associations of PA and SB with MetS among participants of the study. There were 32.1% (95%CI = 30.7, 33.6%) and 27.2% (95%CI = 25.5, 30.1%) of participants with insufficient and sufficient PA, separately, experienced MetS. And, 31.7% (95%CI = 30.4, 33.0%) and 21.6% (95%CI = 21.5, 28.0%) of subjects with prolonged and shortened SB had MetS, respectively. After adjustment for potential confounding factors, subjects with sufficient PA were less likely (OR = 0.77, 95%CI = 0.67, 0.88) to experience MetS, independently of SB, relative to their counterparts with insufficient PA, while a lower odds (OR = 0.74; 95%CI = 0.61, 0.89) of experiencing MetS was examined for participants with shortened SB, also independently of PA, compared to those with prolonged SB in the study.

**Table 4:** The separate and joint association of PA, SB and MetS in urban men aged 60+ years in regional China.

		**Explanatory variable**		**Prevalence of MetS ^a^**	**OR (95%CI) for experiencing MetS** ^**b**^					
		**PA^c^**	**SB^d^**	**% (n/N)**	**Model 1^†^**			**Model 2^‡^**		
**Overall**
	Separate	Insufficient		32.1 (1,295/4035)	1			1		
		Sufficient		27.2 (404/1,485)	0.79 (0.69, 0.90)			0.77 (0.67, 0.88)		
			Prolonged	31.7 (1,529/4,830)	1			1		
			Shortened	24.6 (170/690)	0.71 (0.59, 0.85)			0.74 (0.61, 0.89)		
	Joint	Insufficient	Prolonged	32.9 (1,150/3,500)	1			1		
		Insufficient	Shortened	27.1 (145/535)	0.76 (0.62, 0.93)	1		0.81 (0.65, 0.99)	1	
		Sufficient	Prolonged	28.5 (379/1330)	0.81 (0.71, 0.94)		1	0.80 (0.70, 0.92)		1
		Sufficient	Shortened	16.1 (25/155)	0.39 (0.26, 0.61)	0.52 (0.32, 0.83)	0.48 (0.31, 0.75)	0.41 (0.26, 0.63)	0.48 (0.29, 0.77)	0.47 (0.30, 0.75)

MetS: metabolic syndrome.

a MetS was identified according to diagnostic criteria recommended by Chinese Diabetes Society.

b OR: odds ratio; CI: confidence interval.

c Physical activity was categorized into “insufficient PA (<150 min/week)”and “sufficient PA (≥150 min/week)” based on weekly moderate physical activity time.

d Sedentary behavior was classified as “shortened SB time (<2 h/day)” or “prolonged SB time (≥2 h/day)” according to SB recommendations for Chinese people.

† Model 1 was an unadjusted mixed-effect logistic regression model with PA as the single predictor and adjustment for neighborhood-level clustering effects.

‡ Model 2 was a multivariate mixed-effect logistics regression model with adjustment for age, educational attainment, meat consumption, smoking, drinking, physical activity (where applicable),sedentary time (where applicable), history of hypertension and diabetes and neighborhood-level clustering effects.

Among participants with shortened SB, these with sufficient PA were less likely to experience MetS relative to the counterparts with insufficient PA (OR = 0.48; 95%CI = 0.29, 0.77). On the other hand, for subjects with sufficient PA, those with shortened SB than their counterparts with prolonged SB were also less likely to have MetS (OR = 0.47; 95%CI = 0.30, 0.75). Furthermore, compared to participants in the sub-group of insufficient PA and prolonged SB, those either within categories of insufficient PA and shortened SB (OR = 0.81; 95%CI = 0.65, 0.99), sufficient PA and prolonged SB (OR = 0.80; 95%CI = 0.70, 0.92), or sufficient PA and shortened SB (OR = 0.41; 95%CI = 0.26, 0.63) were at significantly lower risk to experience MetS, respectively.

## Discussion

The main purposes of this population-based study were to investigate the separate and combined relationship between PA, SB and MetS among urban men aged 60+ years in regional China. It was found that PA was in a positive and SB in a negative relation to MetS. Moreover, a joint association was observed that sufficient PA and shortened SB might exert an additive influence on MetS. Further, the association of PA with MetS and the link between SB and MetS were independent of each other.

Our findings regarding the individual associations of PA and SB with Mets were in line with previous studies conducted among people aged either 60+ or 18+ years in both developed countries and developing societies including China (10, 13–18, 33). This suggests that the separate relationships between PA, SB and MetS are strongly consistent among adults with different ages and from societies with different cultural and social contexts worldwide. Meanwhile, in our study, it was also observed that sufficient PA might predict lower odds of MetS even for participants with shortened SB, and, shortened SB could reduce the likelihood of MetS for those with sufficient PA. This implies that sufficient PA and shortened SB can not substitute for one another regarding their individual association with MetS. A recent study conducted among adults aged 65 years in Canada reported that, even for those extremely active people, increased SB was also significantly associated with an elevated risk of MetS (34). This adds further evidence that PA promotion and/or SB reduction are in significantly favorable relation to Mets for people irrespective of their present status of SB and/or PA. Thus, it is of public health importance that campaigns on PA promotion and/or SB reduction are beneficial for general population without necessary consideration of residents' status of SB and PA.

Another major finding of our study was that sufficient PA and shortened SB might exert additive influence on MetS among urban elderly men in regional China. Compared to the reference people who were with insufficient PA and prolonged SB, participants with either insufficient PA and shortened SB or sufficient PA and prolonged SB were at significantly lower risk of MetS, while those subjects with concurrent sufficient PA and shortened SB were at just a half-fold likelihood of MetS. One study using the data from 2009 and 2015 China Health and Nutrition Surveys (CHNS) also identified a joint association of PA and SB with MetS among adults aged 18+ years in China (33). However, in this CHNS study, participant's PA level was calculated based on the sum of occupational, domestic and leisure-time physical activities (33), which was different from that only leisure-time PA was used in our study. Similar findings on joint associations of PA and SB with MetS were also reported from studies conducted among adults in Brazil and Denmark (35, 36). MetS was diagnosed using the NCEP-ATP III criteria, and PA was assessed with a semi-quantitative scale, the Baecke questionnaire, among adults aged 50+years in the Brazilian study (35). For the study conducted among Danish subjects with a mean age of 52 years, MetS was measured with IDF criteria using non-fasting venous blood samples, and PA referred to self-reported average level of leisure-time PA over the past year (36). Consistent evidence from different studies suggests that such an additive relationship between PA, SB and MetS may hold widely among adults with different age and from different societies.

There are some epidemiological and physiological explanations on the relationship between PA, SB and MetS. Based on population-level epidemiological evidence, it has been well-proved worldwide that PA was negatively and SB was positively associated with all the five components of MetS (37, 38). Thus, it is plausible that PA and SB exert influence on MetS through its components, which reasonably results in a consistent direction of associations of PA and SB with MetS and its components. This may, at least partly, explain the negative relationship between PA and MetS as well as the positive association of SB with MetS.

Moreover, there may be several potential physiological mechanisms behind the relationship between PA, SB and MetS. PA is a major pathway of energy expenditure, and it benefits energy balance through not only short-term caloric expenditure (39) but also long-term energy expenditure such as changes in muscle structure, an increase in mitochondria in fiber and secretion of metabolically beneficial hormone, and a decrease in postprandialhepatic lipogenesis (40). On the other hand, prolonged SB may cause a reduction in levels of glucose transporter 4 (GLUT 4) in skeletal muscles (41) and a decrease in insulin sensitivity (42). Muscle lipoprotein lipase, an enzyme that hydrolyzes circulating triglyceride-rich lipoproteins, is highly sensitive to SB (43). Thus, prolonged SB may reduce the activity of muscle lipoprotein lipase and consequently increases plasma triglyceride levels through reducing triglyceride hydrolysis (43). Therefore, this may partially explained the associations of PA and SB with MetS from the perspective of physiology.

This study has important public health implications for future campaigns of population-based MetS prevention among elderly people in China. For those residents aged 60+, they get retired and thus have no job to do. Consequently, they have so much discretionary leisure time. Therefore, the problem is that retirees not only have sufficient discretionary time to do PA but also to engage in SB. The challenge for elderly people to prevent MetS is to spend sufficient time in moderate/vigorous PA and, in the meantime, to spend the shorter time in SB the better. Major findings from our study demonstrated that PA and SB individually exerted opposite effect on MetS independently of each other, and moreover sufficient PA and shortened SB together exerted additive favorable influence on MetS. These strongly suggest that regularly concurrent PA promotion and SB reduction shall be encouraged for elderly people to prevent MetS from the perspective of public health.

Several strengths of this study are worthy of being mentioned. First, study participants were representative of general urban elderly men in a typical mega-city in China. Moreover, a high response rate (95.3%) implies generalizability of study findings. Second, an ethnicity-specific definition of MetS was used to identify MetS, which warranted a tailored diagnosis of MetS for Chinese participants in the study. Third, stratified and cross-over analysis approaches were applied to examine individual and joint associations of PA and SB with MetS. The last, interesting findings, not only an independently separate relationship between PA, SB and MetS but also an additively joint association of sufficient PA and shortened SB with MetS were observed.

There are also some limitations in this study. Firstly, data on PA and SB were self-reported by participants, although the instrument, IPAQ, has been validated for Chinese people. Secondly, no causality of the relationships between PA, SB and MetS could be inferred due to the cross-sectional nature of our study. Thirdly, the CDS definition of MetS included an option of 2-h plasma glucose level ≥7.8 mmol/L as elevated blood glucose. However, 2-h plasma glucose was not assessed for participants, which might result in an under-estimation of MetS prevalence in this study. Finally, due to only one subject with sufficient PA and shortened SB among sub-group of older-old participants aged 80+ years, stratified analysis by age was not appropriate in this study. Therefore, findings reported in the present study need to be interpreted with prudence. In future, longitudinal observational studies and population-based intervention trials are welcome to examine the potential causal associations of PA and SB with MetS, and to assess the impact of PA promotion and SB reduction on MetS among not only urban elderly men but also other subgroups of population in China.

## Conclusions

PA was negatively associated with MetS, and SB was positively linked to MetS among urban men aged 60+ years in regional China. The PA-MetS and SB-MetS relationships were independent of each other. Moreover, sufficient PA and shortened SB might exert additively joint influence on MetS. This study has important public health implications that concurrent regular PA promotion and SB reduction need to be recommended for people with the purpose to optimize the effectiveness of MetS prevention.

## Data availability statement

The data included in the manuscript are available from the corresponding author FX, upon reasonable request.

## Ethics statement

The studies involving human participants were reviewed and approved by the Ethics Committee of Geriatric Hospital of Nanjing Medical University. The patients/participants provided their written informed consent to participate in this study.

## Author contributions

SG and FX conceived and designed the present study. QL, HW, GL, and FX are responsible for data acquisition. FX analyzed the data presented in this manuscript. SG obtained financial support for the present work. QL, HW, GL, YH, QY, SG, and FX wrote and critically reviewed the manuscript. All author approved the final version for submission and was also responsible for all aspects of the work presented in this manuscript.

## Funding

This present work was supported by a key grant from the foundation of Nanjing Institute of Physical Education and Sports (JSCIC-MP21001).

## Conflict of interest

The authors declare that the research was conducted in the absence of any commercial or financial relationships that could be construed as a potential conflict of interest.

## Publisher's note

All claims expressed in this article are solely those of the authors and do not necessarily represent those of their affiliated organizations, or those of the publisher, the editors and the reviewers. Any product that may be evaluated in this article, or claim that may be made by its manufacturer, is not guaranteed or endorsed by the publisher.
